# Revisiting Robustness and Evolvability: Evolution in Weighted Genotype Spaces

**DOI:** 10.1371/journal.pone.0112792

**Published:** 2014-11-12

**Authors:** Raghavendran Partha, Karthik Raman

**Affiliations:** 1 Department of Biotechnology, Bhupat and Jyoti Mehta School of Biosciences, Indian Institute of Technology Madras, Chennai, Tamil Nadu, India; University of California Irvine, United States of America

## Abstract

Robustness and evolvability are highly intertwined properties of biological systems. The relationship between these properties determines how biological systems are able to withstand mutations and show variation in response to them. Computational studies have explored the relationship between these two properties using neutral networks of RNA sequences (genotype) and their secondary structures (phenotype) as a model system. However, these studies have assumed every mutation to a sequence to be equally likely; the differences in the likelihood of the occurrence of various mutations, and the consequence of probabilistic nature of the mutations in such a system have previously been ignored. Associating probabilities to mutations essentially results in the *weighting* of genotype space. We here perform a comparative analysis of weighted and unweighted neutral networks of RNA sequences, and subsequently explore the relationship between robustness and evolvability. We show that assuming an equal likelihood for all mutations (as in an unweighted network), underestimates robustness and overestimates evolvability of a system. In spite of discarding this assumption, we observe that a negative correlation between sequence (genotype) robustness and sequence evolvability persists, and also that structure (phenotype) robustness promotes structure evolvability, as observed in earlier studies using unweighted networks. We also study the effects of base composition bias on robustness and evolvability. Particularly, we explore the association between robustness and evolvability in a sequence space that is AU-rich – sequences with an AU content of 80% or higher, compared to a normal (unbiased) sequence space. We find that evolvability of both sequences and structures in an AU-rich space is lesser compared to the normal space, and robustness higher. We also observe that AU-rich populations evolving on neutral networks of phenotypes, can access less phenotypic variation compared to normal populations evolving on neutral networks.

## Introduction

Biological systems display resilience to perturbations, which are often mutations. Mutations occur at the level of genotype and the resultant changes are observed at the level of phenotype. Depending on the level of complexity, the definitions of genotype and phenotype change. For instance, the amino acid sequence of a protein could be its genotype while phenotype can be defined by its structure, whereas in a metabolic pathway, changes at the enzymatic level (genotype) are reflected in the metabolic capabilities of an organism (phenotype). The resilience of biological systems, or their robustness, is highly intertwined with their *evolvability*, the ability of a system to show variation in its behaviour in response to mutations. These definitions of robustness and evolvability seem to entail a fundamental paradoxical relationship — if a system is highly robust to mutations, then mutations cannot lead to variation, which means less evolvability. However, Wagner [1] showed that robustness and evolvability are not universal properties of a system but can only be clearly defined in the context of a genotype or phenotype, and in doing so, the apparent inherent paradoxical relationship between robustness and evolvability can be resolved. The biological system of choice in the study was the *genotype network* of RNA sequences and their secondary structures [1]. The genotype space is given by the set of RNA sequences, and two sequences (genotypes) are connected, or are neighbours, if they differ by a single point mutation, *i.e.* they differ by a single nucleotide. The phenotype space is given by the set of secondary structures corresponding to the genotypes (sequences). Neutral neighbours are genotypes that are neighbours and have the same phenotype. The set of genotypes that are connected and have the same phenotype comprise a *neutral network*. For such a system, the relationship between robustness and evolvability has been explored and it is observed that genotype robustness and evolvability display an inverse relationship, whereas phenotype robustness and evolvability, on the contrary, show a positive correlation. A major assumption in this and many similar studies [2], [3] is that every mutation is equally likely. In other words, every neighbour of a node in the genotype space can be reached with equal likelihood.

The mutations in question in this system are single nucleotide changes, which are either transitions or transversions. Transitions are intra-purine or intra-pyrimidine conversions (viz. A<->G and C<->T), while transversions are conversions from purines to pyrimidines and vice-versa (viz. A<->C, A<->T, G<->C, G<->T). Transitions, although fewer in number, have a higher incidence compared to transversions [4], and their relative rates of occurrence are given by the transition–transversion ratio, which is denoted by the term kappa (κ). In the genotype space of RNA sequences, an edge corresponds to a single point mutation and hence, depending on the type of mutation, each mutation can be associated with a probability of occurrence, which can be inferred from the transition–transversion ratio. Associating probabilities to the edges essentially results in ‘weighting’ of the space, such that some edges have higher weights (mutations that are more probable) compared to others. Contemporary studies explore the relationship between robustness and evolvability assuming an equal likelihood for the occurrence of all types of mutations [1]–[3]. A theoretical framework that incorporates mutational probabilities according to observed transition–transversion ratios allows a more accurate portrayal of biological reality. In this study, we choose neutral networks of RNA sequences as the biological system and examine how the estimation of the system properties such as robustness and evolvability change upon weighting the genotype space. We subsequently explore the relationship between robustness and evolvability of the system.

## Results

The genotype space is given by the set of all possible sequences. For sequences of length *n*, the genotype space consists of *4^n^* sequences. The phenotype space which is the set of secondary structures of the genotypes roughly corresponds to *1.8^n^* structures [5], [6]. Neutral neighbours are genotypes which differ by a single nucleotide and have the same phenotype. Therefore, a neutral network of a phenotype is given by the set of connected sequences (genotypes) which form the same structure (phenotype) [7]. The 1-neighbourhood of a neutral network is the set of all genotypes that are a single mutation away from the genotypes that form the neutral network.

### Genotype and phenotype robustness and evolvability

In order to define robustness and evolvability, we build upon the definitions put forth by Wagner [1]. Assigning probabilities or weights to the edges of the neutral network will change the associated probabilities of mutation; hence, the changes need to be reflected in the definitions of robustness and evolvability. In order to incorporate this *weighting* of the genotype space, we modified the definitions as follows:

#### Genotype robustness and evolvability

Previously, genotype robustness *R_G_* was the fraction of neutral neighbours of a given genotype ***G***. Assigning a probability to each edge (mutation) in the neutral network will mean evolving to different neutral neighbours will have different probabilities. To account for this, we define genotype robustness *R_G_* as the probability of reaching a neutral neighbour via a single mutation. Genotype evolvability *E_G_* of a sequence ***G*** with phenotype ***P*** was originally considered as the fraction of unique phenotypes (structures different from ***P***) in the 1-neighbourhood of the genotype [1]. Instead, we define *E_G_* as the summation of the mean probabilities of evolving to a structure different from ***P***, in the 1-neighbourhood of G. If we consider all mutations to be equally likely, this definition reduces to the earlier definition of genotype evolvability (refer Text S1 for a more elaborate discussion).

#### Phenotype robustness and evolvability

Phenotype robustness *R_P_* was earlier defined [1] as the fraction of neutral neighbours of a genotype averaged over all the genotypes ***G*** with a given phenotype ***P***. This essentially is the mean genotype robustness of all the genotypes ***G*** with a given phenotype ***P***. As we have changed the definition of genotype robustness, this change will also be reflected in the definition of phenotype robustness. For a given phenotype, the number of unique structures in its 1-neighbourhood was considered as a measure of phenotype evolvability *E_P_*. Here, we consider the mean probability of evolving from the given phenotype ***P*** to a different phenotype summed over all observed different phenotypes in the 1-neighbourhood, as the definition of phenotype evolvability (refer Text S1 for details).

### Weighting the network changes robustness and evolvability

We estimated the genotype robustness and evolvability for this genotype space of 10^6^ sequences. The genotype space is weighted using three different values of transition–transversion ratio (κ) vis-à-vis 0.5, 2.5 and 10. The transition–transversion ratio observed across genomes, usually lies in the range 2.1 to 2.8 [8], [9]. In our study, we consider a value of κ = 2.5 and in addition, we also consider an extreme value of κ = 10. We perform a comparative analysis of the results observed for these weighted networks (κ = 2.5 and κ = 10) with the results of an unweighted network (κ = 0.5).

We computed the genotype robustness and evolvability using the above definitions and found that as the value of κ increases, there is an increase in the value of average value of genotype robustness for the set of 10^6^ sequences. On the other hand, the average sequence evolvability decreases with increase in κ (Table 1).

**Table 1:** Mean genotype robustness and evolvability of 10^6^ sequences (genotypes).

κ	Mean genotype robustness	Mean genotype evolvability	Spearman rank correlation	
			*r*	*p*
0.5	0.42	0.28	−0.747	<10^−17^
2.5	0.48	0.25	−0.756	<10^−17^
10	0.50	0.24	−0.758	<10^−17^

The genotype space was weighted using three different values of κ. We observed that with increasing κ, the mean genotype robustness increases while mean genotype evolvability decreases. In a pair-wise Wilcoxon signed rank test for all pairs of datasets, the *p*-values were less than 10^−17^. Spearman rank correlation values mentioned are between genotype robustness and genotype evolvability.

### High sequence robustness corresponds to low sequence evolvability

**Figure 1:**
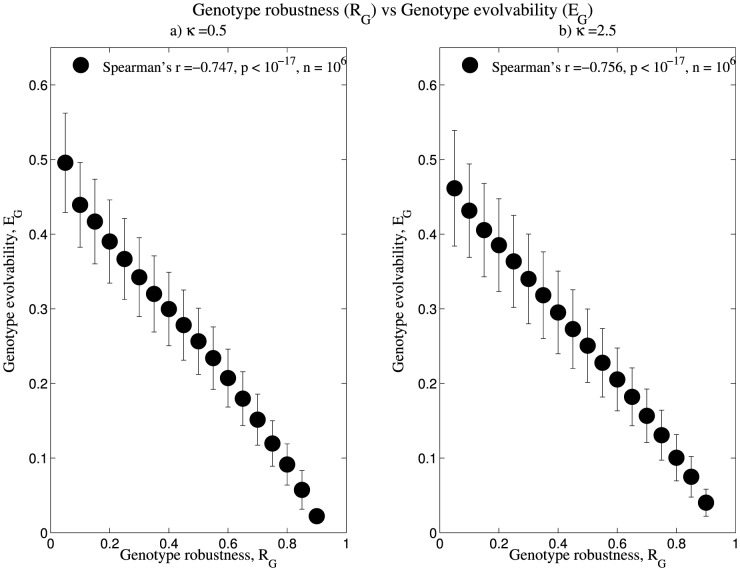
High genotype robustness corresponds to low genotype evolvability. The data shown are based on 10^6^ sequences, whose structures range over three orders of frequency. The genotype space was weighted using κ = 0.5 and 2.5. Length of the error bars correspond to one standard error of the mean, calculated for 18 bins of data grouped according to R_G_. Refer Figure S1 for data corresponding to κ = 10.

Upon calculating the values of sequence robustness and evolvability, we computed the correlation between these values using Spearman's rank correlation test. We observed that a strong negative correlation persists even upon increasing the value of κ (Figure 1, Figure S1). This result is in agreement with previous studies where negative correlation was observed between genotype robustness and evolvability for an ‘unweighted’ genotype space [1]. We observed that for the genotype space we considered, the average robustness of the genotypes increases while the average evolvability decreases, thus strengthening the inverse relationship between the two. Further, the magnitude of negative correlation increases as the κ value increases.

### High structure robustness corresponds to high structure evolvability

Since neutral networks of phenotypes are vast in size, it is not possible to exactly determine the values of phenotype robustness and evolvability as defined in the previous section. This is because identifying *all* genotypes that form a given phenotype is not feasible, even for phenotypes that occur only once in the genotype space [1]. Hence, we adopted a random sampling approach, whereby we inversely folded a set of sequences for each structure (phenotype) and for this set we computed the structure (phenotype) robustness *R_P_* and evolvability *E_P_*. However, in order to account for the size of the neutral network of the phenotype, the evolvability of the phenotype was multiplied by the frequency of occurrence of the phenotype, which is a proxy for the size of the neutral network [1]. Hence, this final entity was considered as the phenotype evolvability *E_P_*.

**Figure 2:**
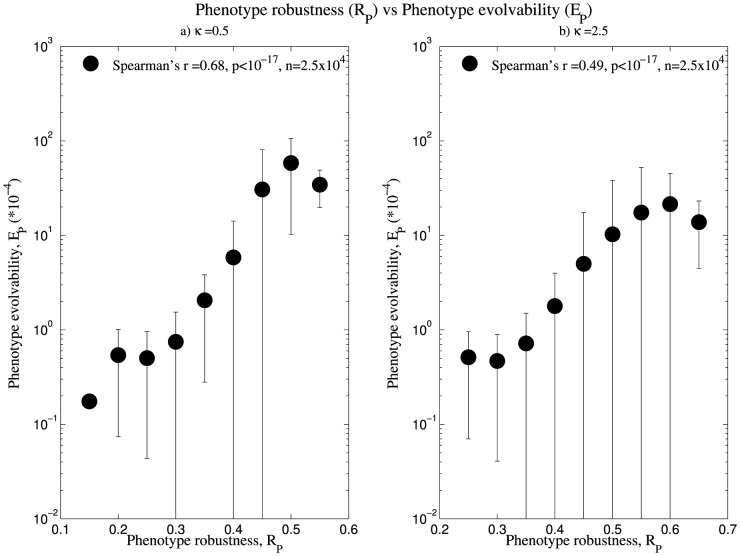
High phenotype robustness corresponds to high phenotype evolvability. The data shown are based on 2.5×10^4^ structures, whose frequency spans three orders of magnitude, and for 100 inversely folded sequences for each structure. The neutral networks corresponding to these structures was weighted using κ = 0.5 and 2.5. Length of the error bars correspond to one standard error of the mean, calculated for 9 bins of data grouped according to R_P_. Refer Figure S2 for data corresponding to κ = 10.

On calculating the phenotype robustness and evolvability for 2.5×10^4^ phenotypes, we observed that phenotype robustness for a given phenotype increases with increasing κ value, while an opposite trend is seen for phenotype evolvability (Table 2). We subsequently obtained the correlation between these values using the Spearman's rank correlation test. We observed a positive correlation in all the cases, with a decrease in the correlation as the κ value increased (Figure 2, Figure S2).

**Table 2:** Mean phenotype robustness and evolvability of 2.5×10^4^ structures (phenotypes).

κ	Mean phenotype robustness	Mean phenotype evolvability (*10^−4^)	Spearman rank correlation	
			*r*	*p*
0.5	0.32	4.40	0.68	<10^−17^
2.5	0.39	3.96	0.49	<10^−17^
10	0.42	3.73	0.39	<10^−17^

The neutral networks of these phenotypes were weighted using three different values of κ. We observed that with increasing κ, the mean phenotype robustness increases while mean phenotype evolvability decreases (in a pair-wise Wilcoxon signed rank test for all pairs of data sets, the *p*-values were less than 10^−17^). Spearman rank correlation values mentioned are between phenotype robustness and phenotype evolvability.

### More robust phenotypes can access more variation via population evolution

Starting from a given genotype (sequence), evolution on the corresponding phenotype's neutral network leads to variation, in the form of new accessible phenotypes, the extent of which is governed by the robustness and evolvability of the phenotype. We performed a comparative analysis using two phenotypes of different frequencies, and hence robustness [1], and observed the variation that these phenotypes can access through evolution on their respective neutral networks. We inversely folded 20 sequences each, for two different structures with frequencies 10^−3^ and 10^−6^, using the Vienna RNA package [10]. For each of these 40 sequences, we established a population of 100 identical sequences. The populations subsequently underwent rounds of mutations at the rate of µ = 1 (one nucleotide per sequence per generation) while ensuring that mutations were neutral. We ensured neutrality by eliminating non-neutral mutants and replacing them with randomly sampled neutral mutants in the population.

At the end of each generation, we calculated the total number of unique structures found in the 1-neighbourhood of the entire population. We also calculated the cumulative number of novel phenotypes that are accessible in the 1-neighbourhood of the population. For this purpose, at the end of each generation, we counted the number of phenotypes that are observed in the 1-neighbourhood of the population, particularly counting those that were not observed in previous generations. This yielded the number of novel phenotypes encountered in that generation. The cumulative number of the novel phenotypes encountered was considered as a measure of the total variation that had been rendered accessible over the generations.

**Figure 3:**
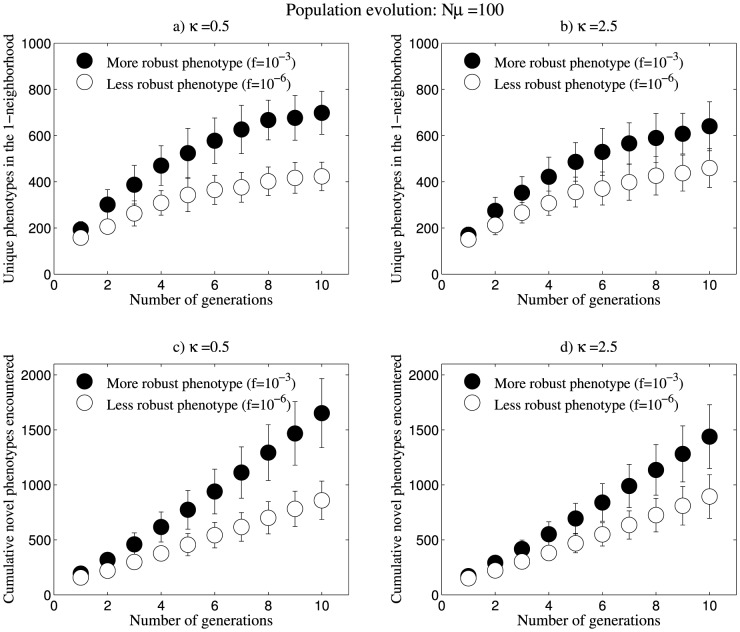
Populations evolving at the rate Nµ = 100. (a), (b) The more robust phenotype has higher structural diversity in its 1-neighbourhood. We observe that the number of unique phenotypes encountered in the 1-neighbourhood by both the phenotypes is higher for κ = 0.5 than κ = 2.5. (c), (d) More robust phenotypes evolving on larger neutral networks have greater access to variation. We observe that the cumulative novel phenotypes encountered by both phenotypes are higher for κ = 0.5 than κ = 2.5. Data shown are for a population of 100 identical sequences for each of 40 inversely folded sequences from the two phenotypes. Mutations occur at the rate of µ = 1 (one nucleotide per sequence per generation). The neutral networks of these phenotypes are weighted using κ = 0.5 and 2.5. Refer Figure S3 for data corresponding to κ = 10.

We performed the above analysis for the three values of κ and found that more robust phenotypes can access more variation even in short time scales such as 10 generations. Also, the cumulative novel phenotypes observed at the end of each generation was consistently higher for the more robust phenotype (Figure 3, Figure S3).

In order to understand if the observed trend was unique to the structures that we used, we performed population evolution at the rate of Nµ = 100 for 10^3^ structures whose frequencies vary over three orders of magnitude. We observe that there is a weak but significant positive correlation between structure frequency and accessible variation in terms of cumulative novel phenotypes encountered at the end of 10 generations. This means that populations evolving on more frequent (and hence robust) phenotypes have more access to phenotypic variation. The degree of weighting does not affect the nature of this relationship but we observe that there is a decrease in the accessibility to variation as we increase the degree of weighting (κ) of the genotype space (see Table S1).

**Figure 4:**
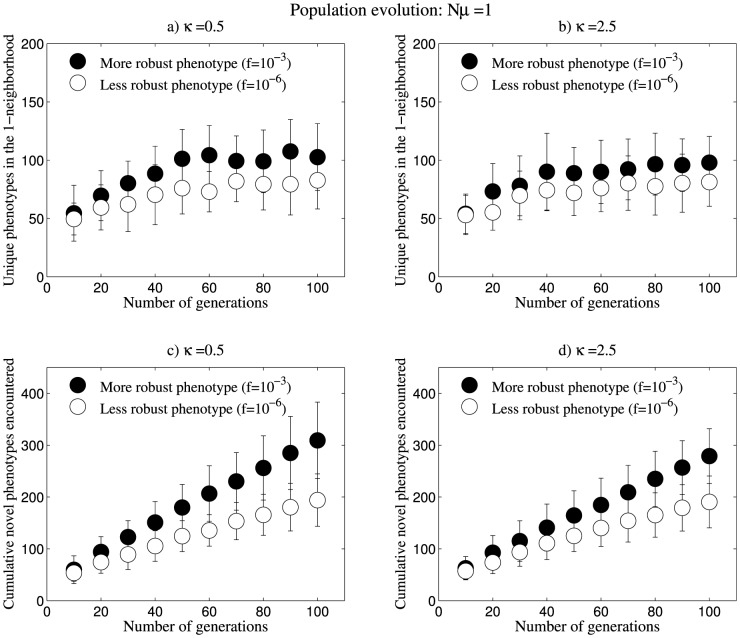
Populations evolving at the rate Nµ = 1. Data shown are for populations of N = 10 identical sequences for each of the 40 inversely folded sequences from the two phenotypes. These sequences are evolving at the rate of µ = 0.1 (one nucleotide per 10 sequences per generation). (a), (b) In populations evolving at the rate of Nµ = 1, the number of unique phenotypes encountered by the more robust phenotype increases at a faster pace compared to the less robust phenotype. The more robust phenotype can access a structurally more diverse 1-neighbourhood compared to the less robust phenotype by the end of 100^th^ generation. (c), (d) More robust phenotypes can access encounter more cumulative novel phenotypes during evolution. We observe that even for low rates of evolution, such as Nµ = 1, more robust phenotypes can access greater variation. The neutral networks of the phenotypes are weighted using κ = 0.5 and 2.5. We observe that both unique phenotypes in 1-neighborhood and cumulative novel phenotypes decrease as we increase the value of κ. We did not observe significant results for κ = 10.

We also analysed the evolutionary dynamics of populations evolving at the rate of Nµ = 1. For this purpose, we took a seed population of N = 10 and performed mutations at the rate µ = 0.1 (one nucleotide per 10 sequences per generation). The dynamics of population evolution changes significantly with a decrease in the value of Nµ [11]–[13] and hence we tried to observe the effects of this change. We found that, even for Nµ = 1, at the end of the 100^th^ generation, the more robust phenotype typically has a more diverse 1-neighbourhood. Also, the cumulative novel phenotypes encountered at the end of the 10^th^ generation is higher for the robust phenotype (Figure 4). We also performed the analysis for 10^3^ structures whose populations were evolving at the rate of Nµ = 1. Here again, we observe that there is a weak but significant positive correlation between structure frequency and accessible variation in terms of cumulative novel phenotypes encountered at the end of 100 generations (see Table S2). Therefore, even for low rates of evolution such as Nµ = 1 populations evolving on more frequent (and hence robust) phenotypes have more access to phenotypic variation. Similar to the results observed for Nµ = 100, we find that degree of weighting does not affect the nature of this relationship, but there is a decrease in the accessibility to variation as we increase the degree of weighting (κ) of the genotype space. However, compared to the previous case (Nµ = 100), we observed a relatively smaller decrease in accessibility to variation with increasing κ.

### Analysis of AU-rich sequence space

The variation in GC content between genomes is a central issue in evolutionary genomics, and is understood to be influenced by variation in selection, mutational bias, and biased recombination-associated DNA repair [14]. A GC pair is bound by three hydrogen bonds, while an AT pair is bound by two hydrogen bonds thereby conferring higher stability to the DNA molecule. The thermal adaptation hypothesis conjectures that as GC pairs in DNA are more thermally stable than AT pairs, high GC content may be a result of a selective pressure to survive in high temperatures [15]. However, this hypothesis has been refuted, after a comparative study of numerous prokaryotes showed that there was no correlation between genomic GC content and growth temperature [16]. However, it was shown in the same study that there was a positive correlation between GC content of structured RNAs (transfer RNAs, ribosomal RNAs) and growth temperatures. GC pairs are much more stable than AU pairs, again owing to the fact that GC pairs are bound by three hydrogen bonds, compared to two bonds in AU pairs, thus explaining the GC bias in RNA structures exhibiting more tolerance to high temperatures [16].

**Figure 5:**
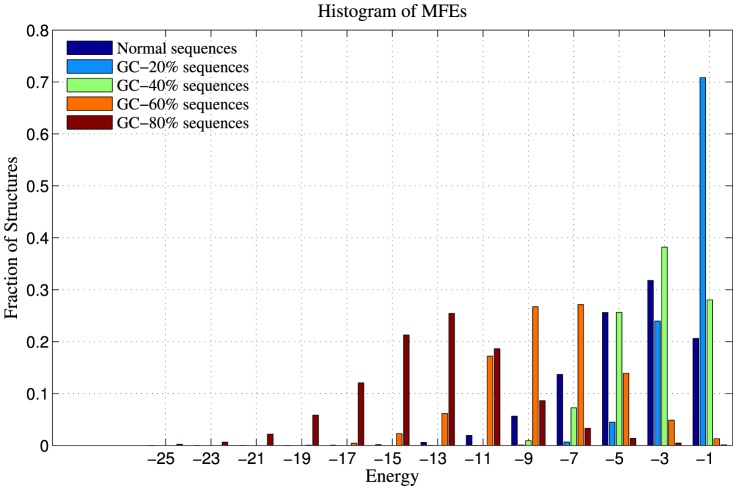
Minimum Free Energies of structures formed by RNA sequences of varied GC content. Data shown are for one million sequences of varied GC-content in comparison to normal unbiased sequences. The MFEs were calculated using viennaRNAFold routine of Vienna RNA package [10]. We observe that the Minimum Free Energies of sequences become less negative as their GC content decreases, reflecting a decrease in thermal stability.

**Figure 6:**
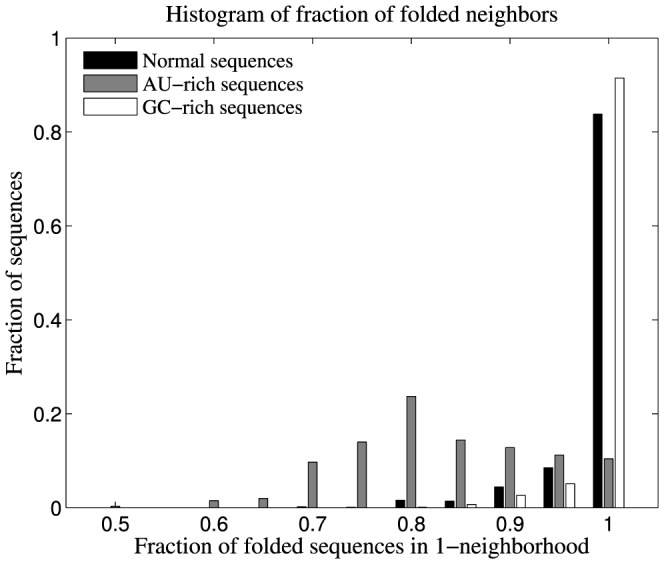
Comparison of 1-neighbourhoods of sequences of varied GC-content. Data shown are the histogram of fractions of folded sequences in 1-neighbourhood for each of 1000 randomly chosen normal, AU-rich (GC content ≤20%) and GC-rich (GC content ≥80%) sequences. We observe that a majority of AU-rich sequences have a lower fraction of folded neighbours, compared to normal sequences and GC-rich sequences, in their 1-neighbourhood. The sequences were folded using viennaRNAFold routine of Vienna RNA package [10].

In an RNA sequence space, we observe that sequences with higher GC content have more negative Minimum Free Energy (Figure 5), as expected. GC-rich sequences (GC content ≥80%), are significantly more stable than normal unbiased sequences. Despite this, we observe that GC-rich sequences have 1-neighbourhoods that are more or less similar to 1-neighbourhoods of normal unbiased sequences (Figure 6), in terms of structure density. On the other hand, an AU-rich sequence space (sequences of GC content ≤20%), is sparsely populated with RNA structures – randomly chosen AU-rich sequences have a significantly lower fraction of folded sequences in their 1-neighbourhoods compared to randomly chosen normal unbiased sequences and GC-rich sequences (Figure 6). Therefore, we restrict our analyses to AU-rich sequence spaces, and try to understand whether base composition bias has an effect on the robustness and evolvability of genotype networks of RNA sequences.

### AU-rich sequences are more robust and less evolvable

**Figure 7:**
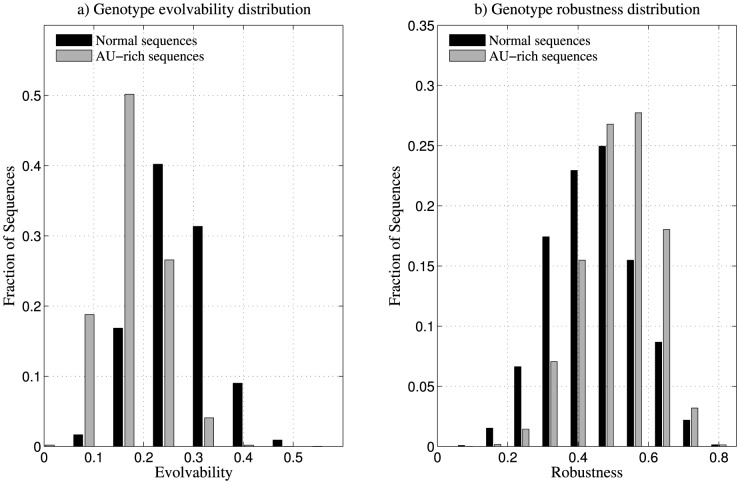
Comparison of genotype robustness and evolvability of normal and AU-rich sequences. (a) Histogram of genotype evolvability of one million normal sequences and one million AU-rich sequences. We observed that a majority of AU-rich sequences have lesser genotype evolvability in comparison to normal sequences. b) Histogram of genotype robustness of one million normal sequences and one million AU-rich sequences. We observed that a majority of AU-rich sequences have higher genotype robustness in comparison to normal sequences. The genotype space is weighted using κ = 2.5.

We computed the genotype robustness and evolvability for 10^6^ AU-rich sequences, and found that AU-rich sequences on average have higher robustness and lower evolvability in comparison to 10^6^ normal sequences (Table 3, Figure 7). The genotype space was weighted using κ = 2.5.

**Table 3:** Mean genotype robustness and evolvability of 10^6^ AU-rich sequences and 10^6^ normal sequences.

Sequence space	Mean genotype robustness	Mean genotype evolvability	Spearman rank correlation	
			*r*	*p*
Normal	0.48	0.25	−0.758	<10^−17^
AU-rich	0.56	0.16	−0.70	<10^−17^

The genotype space was weighted using κ = 2.5. We observed that the mean genotype robustness is higher for AU-rich sequences, while mean genotype evolvability is lesser, in comparison to normal space. In a pair-wise Wilcoxon signed rank test between the two datasets, the *p*-value was less than 10^−8^. Spearman rank correlation values mentioned are between genotype robustness and genotype evolvability.

Although we showed that AU-rich have fewer folded neighbours in their 1-neighbourhoods compared to normal unbiased sequences, we observe that their robustness is higher. Although this seems counter-intuitive, the reason for this observation is that there are more neutral neighbours than non-neutral neighbours for AU-rich sequences, while this is not the case for normal unbiased sequences (see Figure S4). Also, a majority of AU-rich sequences have more neutral neighbours than normal sequences. Upon calculating the values of sequence robustness and evolvability, we computed the correlation between these values using Spearman's rank correlation test. We observed a strong negative correlation between sequence robustness and evolvability for AU-rich sequences (see Figure S5), much like the relationship observed for normal sequences.

### Structure robustness is higher and evolvability is lower in AU-rich space

For our phenotype robustness and evolvability analysis in normal unbiased sequence space, we adopted a random sampling approach, whereby we inversely folded a set of sequences for each structure (phenotype). For this set of sequences, we computed the structure (phenotype) robustness *R_P_* and evolvability *E_P_*. In order to account for the size of the neutral network of the phenotype, the evolvability of the phenotype was then multiplied by the frequency of occurrence of the phenotype. In order to perform a comparative analysis of phenotype robustness and evolvability in AU-rich space and normal space, we need to be able to inversely fold AU-rich sequences for a given structure. In order to do so, we developed a two-step approach to inversely fold AU-rich sequences, which in turn form an AU-rich neutral network (described in detail in Methods)'.

**Figure 8:**
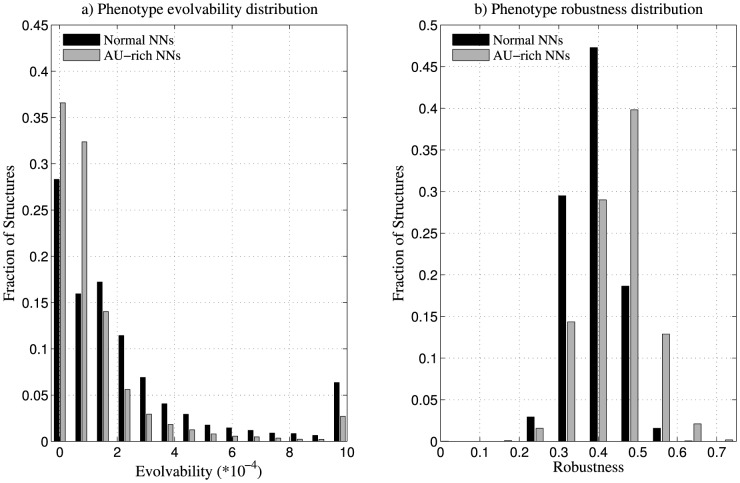
Comparison of phenotype robustness and evolvability of normal and AU-rich sequences. (a) Histogram of phenotype evolvability of AU-rich and normal neutral networks (NNs) of 2.5×10^4^ structures. We observe that AU-rich neutral networks, in general, have lesser phenotype evolvability in comparison to normal neutral networks. (b) Histogram of phenotype robustness of AU-rich and normal neutral networks (NNs) of 2.5×10^4^ structures. We observe that AU-rich neutral networks, in general, have higher phenotype robustness in comparison to normal neutral networks. These neutral networks are weighted using κ = 2.5.

Subsequently, we computed the phenotype robustness and evolvability for AU-rich neutral networks of 2.5×10^4^ structures, comparing the results with that of phenotype robustness and evolvability of normal neutral networks of the structures obtained previously. These networks were weighted using κ = 2.5. We found that AU-rich neutral networks of phenotypes, on an average, have higher robustness, and lower evolvability in comparison to normal neutral networks (Table 4, Figure 8).

**Table 4:** Mean phenotype robustness and evolvability of AU-rich and normal neutral networks of 2.5×10^4^ structures.

Sequence space	Mean phenotype robustness	Mean phenotype evolvability (*10^−4^)	Spearman rank correlation	
			*r*	*p*
Normal	0.39	3.96	0.49	<10^−17^
AU-rich	0.44	1.76	0.69	<10^−17^

These networks were weighted using κ = 2.5. We observed that the mean phenotype robustness is higher for AU-rich neutral networks, while mean phenotype evolvability is lesser, in comparison to normal neutral networks. In a pair-wise Wilcoxon signed rank test between the two datasets, the *p*-value was less than 10^−8^. Spearman rank correlation values mentioned are between genotype robustness and genotype evolvability.

On calculating the values of structure robustness and evolvability, we computed the correlation between these values using Spearman's rank correlation test. We observed a positive correlation between structure robustness and evolvability for AU-rich neutral networks of structures (see Figure S6), similar to the relationship observed for normal neutral networks.

### AU-rich populations evolving on phenotype's neutral network access lesser variation

We contrast the variation accessible to AU-rich populations evolving on a structure's neutral network compared to normal populations evolving on the same neutral network. We note that the populations evolve in different ‘regions’ of the neutral network of the same structure – one region being typically AU-rich and the other being unbiased. Also, in the course of evolving these populations we did not restrict the access of the AU-rich population to AU-rich sequences.

For a phenotype of high robustness (frequency  = 10^−3^), we establish two different starting populations – an AU-rich starting population and a normal starting population as follows: we inversely folded 20 AU-rich sequences and 20 normal sequences, and for each of these 40 sequences, we established a population of 100 identical sequences. We consequently evolved both populations at the rate of µ = 1 (one nucleotide per sequence per generation), under the same conditions employed in our previous population evolution (Nµ = 100) analysis.

**Figure 9:**
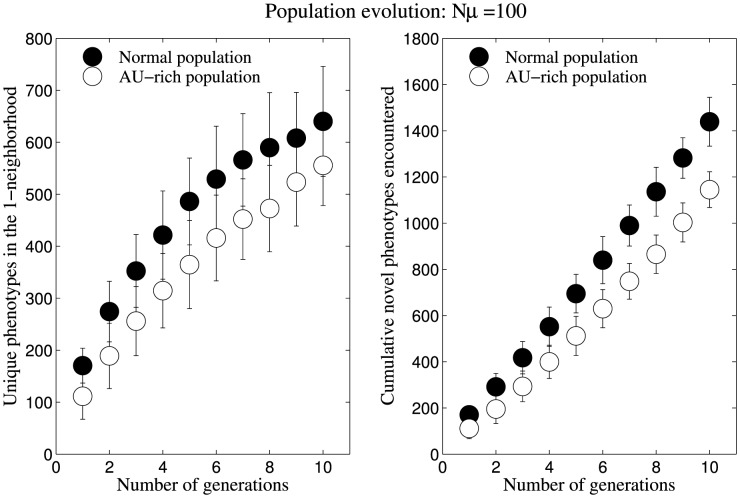
Population evolution on a highly robust phenotype's neutral network with AU-rich and normal starting populations. We observe that AU-rich starting population can access lesser variation: lesser number of cumulative novel phenotypes and unique phenotypes in 1-neighbourhood during 10 generations of mutations. Data shown are for a population of 100 identical sequences for each of 20 inversely folded normal sequences and 20 inversely folded AU-rich sequences for the phenotype. Mutations occur at the rate of µ = 1 (one nucleotide per sequence per generation), thereby leading to a rate of evolution of Nµ = 100.

We observe that in comparison to the normal starting population, the AU-rich starting population can access lesser variation in terms of unique phenotypes in the 1-neighbourhood throughout the 10 generations of mutations. Also, the cumulative number of novel phenotypes in the 1-neighbourhood was consistently lesser for AU-rich starting populations (Figure 9). We confirmed that this observation was not unique to the structure that was used, by repeating the analysis for 10^3^ structures. We also find that, similar to normal populations, there is a weak but significant correlation between variation accessible to AU-rich populations evolving on a structure's neutral network and the structure frequency, hence robustness (see Table S3).

We observed similar results for a comparative analysis of AU-rich and normal populations evolving on a structure's neutral network at the rate of Nµ = 1 (see Figure S7), wherein AU-rich populations can access lesser variation compared to normal populations. We observed a weak but significant correlation between variation accessible and structure frequency (see Table S4). Therefore, the variation accessible to AU-rich populations evolving at fast evolutionary rates such as Nµ = 100, as well as slower rates such as Nµ = 1, is lesser compared to a normal population evolving on the same structure's neutral network.

## Discussion

In this study, we discard a widely considered assumption on the organisation of genotypes spaces, namely, an equal likelihood for all mutations, and revisit the fundamental relationships between robustness and evolvability upon weighting of the genotype space. Herein, we see that assuming equally likely mutations essentially overestimates evolvability and underestimates robustness. We see that the robustness of a given genotype/phenotype is higher for a weighted neutral network than for an unweighted neutral network of the same genotype/phenotype. We observe an opposite trend in the case of evolvability, as with increasing transition-transversion ratio (κ), evolvability decreases for both genotypes and phenotypes. Notwithstanding the changes in these values and hence properties of the system, we see that fundamental relationships between genotype robustness and evolvability and phenotype robustness and evolvability are maintained, even upon weighting of the network. We observe that high sequence robustness corresponds to low sequence evolvability, whereas high phenotype robustness correlates to a high phenotype evolvability, as also seen in previous studies [1]. It follows that a more robust phenotype can thus access more variation by evolving on its neutral network. Even after evolution on the neutral network for short time scales (10 generations), we find that the 1-neighbourhood of a more robust phenotype is more diverse. We also observe that in such short time scales, the cumulative number of different phenotypes that can potentially be accessed, is greater for a more robust phenotype, in accordance with earlier work by Wagner [1]. However, we observe that the extent of this accessible variation is overestimated if we ignore the weighting of the network.

We further analysed the effects of base composition bias in RNA sequences on robustness and evolvability. Specifically, we analysed RNAs that have very high AU content; AU-rich sequences essentially form an ‘extreme’ section of the RNA sequence space. Such RNA sequence elements (AU-rich elements or AREs) are usually found in 3′ untranslated region of many messenger RNAs that code for proto-oncogenes, nuclear transcription factors and cytokine [17]. AREs are one of the most common determinants of RNA stability in mammalian cells, and usually target the mRNA for degradation [18]. From our comparative analyses of AU-rich sequence spaces and normal unbiased sequence spaces, we observe that AU-rich genotypes have higher robustness and lesser evolvability. At the phenotype level, we observe that AU-rich neutral networks of phenotypes have higher robustness and lesser evolvability than neutral networks of the same phenotype in normal space. These changes in the values of robustness and evolvability however do not affect the relationship between the properties — genotype robustness and genotype evolvability are negatively correlated in AU-rich spaces, an observation that also holds in normal space. Similarly, phenotype robustness positively correlates to phenotype evolvability in AU-rich spaces, a trend also observed in normal spaces. During evolution on a phenotype's neutral network, phenotypes access lesser variation with a starting population of AU-rich sequences, compared to a starting population of normal sequences. Both structural diversity of 1-neighbourhood and cumulative number of different phenotypes potentially accessible during evolution are lesser when the starting population comprises of AU-rich sequences. This is indicative of the restrictive nature of the AU-rich space.

Overall, we observe two main contributions of our study: first, we have shown that assuming an equal likelihood for all mutations (as in an unweighted network), underestimates robustness and overestimates evolvability of a system. Despite discarding this assumption, we observe that the negative correlation between genotype robustness and genotype evolvability is maintained, and so is the positive correlation between structure (phenotype) robustness and structure evolvability, as observed in earlier studies using unweighted networks. Correspondingly, we also show that populations evolving on neutral networks of phenotypes, weighted or otherwise, can access more phenotypic variation in the case of phenotypes with higher robustness. Secondly, our analyses also shed light on the organisation of an extreme portion of the RNA sequence space, rich in AU bases. Structures in this AU-rich space, while very robust, are able to access lesser variation and are unable to facilitate evolution as well as in the *normal* space. In principle, it may be possible to exploit the relative robustness of AU-rich sequences in designing more mutationally robust RNA molecules. This is also supported by the availability of substantial evidence for the high plasticity of AU-rich sequences, which is their ability to fold into alternative secondary structures with a low energy cost [19], [20]. Previous efforts to understand the effect of compositional bias on the designability of RNA secondary structures [21] and in the adaptive dynamics of RNA populations [22], also show that nucleotide composition affects the evolvability and occurrence of specific structural motifs. Our framework can also be extended to study the effects of mutational bias (towards AU) on robustness and evolvability, considering there is significant evidence that mutation is universally biased towards AT in bacteria [23]. In sum, our approach presents a more extensive characterisation of the RNA genotype space, and the relation between robustness and evolvability in such spaces. It is possible to extend such analyses to other levels of biological organisation, such as metabolic, regulatory or signalling networks, where the importance of the likelihood of different genotype changes may be even more relevant.

## Methods

### Genotype and phenotype space

We chose RNA sequences of length 30 as our genotypes, the same as used in the study by Wagner [1]. This length offers computational tractability while not compromising on the structural diversity of their corresponding phenotypes [1]. We obtain the structure of a given sequence using the routine viennaRNAFold, part of the Vienna RNA package [10]. The resulting structure's representation in the bracket notation allows for ease of comparison between different structures.

### Weighting the neutral network

The 1-neighbourhood of a genotype is the set of all sequences which differ by a single nucleotide from the genotype. Thus, for sequences of length 30, the 1-neighbourhood consists of 90 ( = 3×30) sequences. Weighting of the neutral network involves assigning a probability to each of these (90) mutations based on the value of κ. The transition–transversion ratio (κ) is defined as the ratio of number of transitions and number of transversions. For a given nucleotide (say, A), there are two transversions (A->C and A->T) and one transition (A->G) that are possible. Thus, for a value of κ = 0.5, all mutations (transitions and transversions), have an equal probability. When κ = 1.0, a transition mutation occurs with twice (probability  = 0.5) the probability of each transversion mutation (probability  = 0.25), and similarly κ = 2.5 would mean that transitions would occur with five times (probability  = 0.71) the probability of a transversion (probability  = 0.145). This way, all the 90 mutations are assigned probabilities and subsequently normalised, such that the sum of their probabilities equals unity.

### Choice of sequences and structures

In order to compute the correlation between genotype robustness and evolvability, we first generated 10^6^ random RNA sequences of length 30, whose secondary structures were obtained using the routine viennaRNAFold, part of the Vienna RNA package [10]. The frequency of these structures spans over three orders of magnitude. For this set of sequences, we calculated the genotype robustness and evolvability using the definitions discussed in the Results section and also elaborated in Text S1. Subsequently, we calculated the correlation between the genotype robustness and evolvability using Spearman's rank correlation test.

For the purpose of calculating phenotype robustness and evolvability, we randomly sampled 2.5×10^4^ structures whose frequencies range over three orders of magnitude (10^−6^ to 10^−3^). For each of these structures, we inversely folded 100 sequences using the routine viennaRNAinverse, part of the Vienna RNA package. We estimated the robustness and evolvability in this set of sequences. This value of robustness is a measure of the average robustness in the entire phenotypic space of the given structure. However, it is not appropriate to consider the evolvability arising out of this analysis as the average evolvability of the phenotypic space [24], [25]. This is because the structures in the 1-neighbourhood of this set of sequences are likely to be different from the set of structures observed in the 1-neighbourhood of a different set of sequences that fold into the same structure. Thus, in order to estimate evolvability, we need to identify the unique structures found in these different 1-neighbourhoods and then compute the actual value of evolvability using the definitions provided in Text S1. Owing to computational limitations, we instead consider the product of evolvability in one set of inversely folded sequences and the frequency of the structure in phenotype space as an estimate of phenotype evolvability of the structure [1]. We employ a brute force approach of sampling a large number of sequences (10^12^), and estimate the frequency of a given structure as the fraction of sequences in this sample that fold into the structure. We note that this type of a sampling approach gives an acceptable measure of a structure's frequency, and hence persist with it in the face of better and computationally more expensive algorithms that can precisely estimate the frequency of structures [26]. Upon estimating phenotype robustness and evolvability for these 2.5×10^4^ structures, we obtained the correlation between them using Spearman's rank correlation test.

### Analysis of AU-rich sequence space

We generated 10^6^ random sequences of length 30, with an AU content of at least 80% for our genotype level analysis. The secondary structures of these sequences, the corresponding Minimum Free Energies (MFEs) of the secondary structures were computed using the viennaRNAFold routine of the Vienna RNA package [10]. Genotype robustness and genotype evolvability of these sequences were calculated according to definitions outlined in Text S1.

### Inversely folding AU-rich sequences

In order to perform phenotype level analysis in an AU-rich space, we developed a custom approach to inversely fold AU-rich sequences for a given RNA structure. This was necessitated by the fact that there are no conventional algorithms that can generate a set of AU-rich sequences which fold into a given RNA secondary structure.

We employed a two-step approach to inversely fold AU-rich sequences for a given RNA secondary structure. In the first step, we used the viennaRNAinverse routine part of the Vienna RNA package [10] to inversely fold a population of normal sequences that fold into the given secondary structure. In the second step, we performed a structure-preserving random walk from every sequence in the population towards AU-rich spaces; at each step of the walk, we only accept mutations that do not decrease the AU content of the sequence. We terminate the random walk upon encountering an AU-rich sequence (AU content of ≥80%) that folds into the given secondary structure. Thus, for a given RNA secondary structure, we were able to generate a population of AU-rich sequences which fold into the secondary structure. This population of AU-rich sequences was used for further robustness and evolvability calculations.

## Supporting Information

Figure S1 **High genotype robustness corresponds to low genotype evolvability**. The data shown are based on 10^6^ sequences, whose structures range over three orders of frequency. The neutral network of these structures was weighted using κ = 10. Length of the error bars correspond to one standard error of the mean, calculated for 18 bins of data grouped according to R_G_.
(EPS)

Figure S2 **High phenotype robustness corresponds to high phenotype evolvability.** The data shown are based on 2.5×10^4^ structures, whose frequency spans three orders of magnitude, and for 100 inversely folded sequences for each structure. The neutral network of these structures was weighted using κ = 10. Length of the error bars correspond to one standard error of the mean, calculated for 9 bins of data grouped according to R_P_.
(EPS)

Figure S3 **More robust phenotypes evolving on larger neutral networks have greater access to variation and have higher structural diversity in their 1-neighbourhood.** Data shown are for a population of 100 identical sequences for each of 40 inversely folded sequences from the two phenotypes. Mutations occur at the rate of µ = 1 (one nucleotide per sequence per generation). The neutral networks of these phenotypes are weighted using κ = 10.
(EPS)

Figure S4 **Number of unfolded, neutral and non-neutral (folded) sequences in 1-neighbourhood for each of 1000 randomly chosen normal, AU-rich sequences.** We observe that a majority of AU-rich sequences have a higher number of neutral neighbours than non-neutral neighbours, and also have more neutral neighbours compared to normal sequences. This contributes to the higher robustness of AU-rich sequences compared to normal sequences. The sequences were folded using viennaRNAFold routine of Vienna RNA package [10].
(EPS)

Figure S5 **High genotype robustness corresponds to low genotype evolvability**. The data shown are based on 10^6^ normal sequences and 10^6^ AU-rich sequences, whose structures range over three orders of frequency. The neutral network of these structures was weighted using κ = 2.5. Length of the error bars correspond to one standard error of the mean, calculated for 18 bins of data grouped according to R_G_.
(EPS)

Figure S6 **High phenotype robustness corresponds to high phenotype evolvability.** The data shown are based on AU-rich neutral networks and normal neutral networks of 2.5×10^4^ structures, whose frequency spans three orders of magnitude. The neutral network of these structures was weighted using κ = 2.5. Length of the error bars correspond to one standard error of the mean, calculated for 9 bins of data grouped according to R_P_.
(EPS)

Figure S7 **Population evolution (at the rate of Nµ = 1) on a highly robust (f = 10^−3^) phenotype's neutral network with two different starting populations: AU-rich and normal.** We observe that AU-rich starting population can access lesser variation: lesser number of cumulative novel phenotypes and unique phenotypes in 1-neighbourhood during 10 generations of mutations. Data shown are for a population of 10 identical sequences for each of 20 inversely folded normal sequences and 20 inversely folded AU-rich sequences for the phenotype. Mutations occur at the rate of µ = 0.1 (one nucleotide per 10 sequences per generation).
(EPS)

Table S1 **Population evolution (at the rate of Nµ = 100) for 10^3^ structures of varied robustness whose neutral networks are weighted using κ = 0.5 and κ = 2.5.**
(DOCX)

Table S2 **Population evolution (at the rate of Nµ = 1) for 10^3^ structures of varied robustness whose neutral networks are weighted using κ = 0.5 and κ = 2.5.**
(DOCX)

Table S3 **Population evolution (at the rate of Nµ = 100) for 10^3^ structures of varied robustness with two different starting populations: AU-rich and normal.**
(DOCX)

Table S4 **Number of cumulative novel phenotypes encountered at the end of 100 generations of mutations (at the rate of Nµ = 1), for 10^3^ structures with two different starting populations: AU-rich and normal.**
(DOCX)

Text S1 **Detailed explanation of the various definitions for genotype and phenotype robustness and evolvabilities. (R_G_, E_G_, R_P_, E_P_).**
(PDF)
