# Supplementary material for: Revisiting Robustness and Evolvability: Evolution in Weighted Genotype Spaces
Source: PLoS One. 2014 Nov 12;9(11):e112792. doi: 10.1371/journal.pone.0112792 (PMC4229248; doi:10.1371/journal.pone.0112792)
Supplement: Table S1 — Population evolution (at the rate of Nµ = 100) for 103 structures of varied robustness whose neutral networks are weighted using κ = 0.5 and κ = 2.5. (DOCX) [file pone.0112792.s008.docx]

# SUPPLEMENTARY TABLE S1

| **Κ** | **Cumulative novel phenotypes** | **Correlation with structure frequency** |
| --- | --- | --- |
| 0.5 | 1089±301 | 0.22 |
| 2.5 | 1013±253 | 0.16 |

**Table S1. Number of cumulative novel phenotypes observed at the end of 10 generations of mutations (at the rate of Nµ = 100)**, for 10^3^ structures whose neutral networks were weighted using κ = 0.5 and κ = 2.5. One inversely folded sequence was used to seed a population size of N = 100 and µ =1. With increasing κ value, we observed a decrease in the accessible variation, in the form of cumulative novel phenotypes encountered in the 1‑neighbourhood, at the end of 10 generations (in a pair-wise Wilcoxon signed rank test between the two data sets, the *p*-value was less than 10^-17^). Correlation values mentioned are Spearman’s *r* values, with their respective *p*-values less than 10^-17^.
