# Supplementary material for: Revisiting Robustness and Evolvability: Evolution in Weighted Genotype Spaces
Source: PLoS One. 2014 Nov 12;9(11):e112792. doi: 10.1371/journal.pone.0112792 (PMC4229248; doi:10.1371/journal.pone.0112792)
Supplement: Table S2 — Population evolution (at the rate of Nµ = 1) for 103 structures of varied robustness whose neutral networks are weighted using κ = 0.5 and κ = 2.5. (DOCX) [file pone.0112792.s009.docx]

# SUPPLEMENTARY TABLE S2

| **κ** | **Cumulative novel phenotypes** | **Correlation with structure frequency** |
| --- | --- | --- |
| 0.5 | 244±63 | 0.22 |
| 2.5 | 240±58 | 0.13 |

**Table S2. Number of cumulative novel phenotypes observed at the end of 100 generations of mutations (at the rate of Nµ =1)**, for 10^3^ structures whose neutral networks were weighted using κ = 0.5 and κ = 2.5. One inversely folded sequence was used to seed a population size of N = 10 and µ = 0.1. With increasing κ value, we observed a modest decrease in the accessible variation, in the form of cumulative novel phenotypes encountered in the 1‑neighbourhood, at the end of 100 generations. In a pair-wise Wilcoxon signed rank test between the two data sets, the *p*-value was less than 10^-3^. Correlation values mentioned are Spearman’s *r* values, with their respective *p*-values less than 10^-4^.
