# Supplementary material for: Revisiting Robustness and Evolvability: Evolution in Weighted Genotype Spaces
Source: PLoS One. 2014 Nov 12;9(11):e112792. doi: 10.1371/journal.pone.0112792 (PMC4229248; doi:10.1371/journal.pone.0112792)
Supplement: Table S3 — Population evolution (at the rate of Nµ = 100) for 103 structures of varied robustness with two different starting populations: AU-rich and normal. (DOCX) [file pone.0112792.s010.docx]

# SUPPLEMENTARY TABLE S3

| **Sequence space** | **Cumulative novel phenotypes** | **Correlation with structure frequency** |
| --- | --- | --- |
| Normal | 1013±253 | 0.16 |
| AU-rich | 774±189 | 0.21 |

**Table S3. Number of cumulative novel phenotypes encountered at the end of 10 generations of mutations (at the rate of Nµ =100)**, for 10^3^ structures with two different starting populations. One inversely folded AU-rich sequence and one inversely folded normal sequence were respectively used to seed the two populations of size of N = 100 and µ =1. The neutral networks of these structures were weighted using κ = 2.5. AU-rich populations access less variation compared to normal populations. The *p*-value of pair-wise Wilcoxon signed rank test between the two datasets was less than 10^-17^. Correlation values are Spearman’s *r* values with all *p*-values less than 10^-17^.
