# Supplementary material for: Revisiting Robustness and Evolvability: Evolution in Weighted Genotype Spaces
Source: PLoS One. 2014 Nov 12;9(11):e112792. doi: 10.1371/journal.pone.0112792 (PMC4229248; doi:10.1371/journal.pone.0112792)
Supplement: Table S4 — Number of cumulative novel phenotypes encountered at the end of 100 generations of mutations (at the rate of Nµ = 1), for 103 structures with two different starting populations: AU-rich and normal. (DOCX) [file pone.0112792.s011.docx]

# SUPPLEMENTARY TABLE S4

| **Sequence space** | **Cumulative novel phenotypes** | **Correlation with structure frequency** |
| --- | --- | --- |
| Normal | 248±58 | 0.13 |
| AU-rich | 181±46 | 0.15 |

**Table S4. Number of cumulative novel phenotypes encountered at the end of 100 generations of mutations (at the rate of Nµ =1)**, for 10^3^ structures with two different starting populations. One inversely folded AU-rich sequence and one inversely folded normal sequence were respectively used to seed the two populations of size of N = 10 and µ = 0.1. The neutral networks of these structures were weighted using κ = 2.5. AU-rich populations access less variation compared to normal populations. The *p*-value of pair-wise Wilcoxon signed rank test between the two datasets was less than 10^-17^. Correlation values are Spearman’s *r* values with all *p*-values less than 10^-4^.
