# Supplementary material for: Revisiting Robustness and Evolvability: Evolution in Weighted Genotype Spaces
Source: PLoS One. 2014 Nov 12;9(11):e112792. doi: 10.1371/journal.pone.0112792 (PMC4229248; doi:10.1371/journal.pone.0112792)
Supplement: Text S1 — Detailed explanation of the various definitions for genotype and phenotype robustness and evolvabilities. (RG, EG, RP, EP). (PDF) [file pone.0112792.s012.pdf]

SUPPLEMENTARY TEXT S1

Definitions

In this section, we rationalize the need for new definitions of robustness and evolvability at the level of both genotype and phenotype, so as to incorporate the weighting of the network. We show that the new definitions essentially concur with the earlier definitions put forth by Wagner [1], for an unweighted network.

Let us consider an unweighted network with all edges having the same probability (p=0.33). The genotype space consists of 6 nodes (G1, G2 ... G6) and the phenotype space is given by P1, P2, P3 and P4.

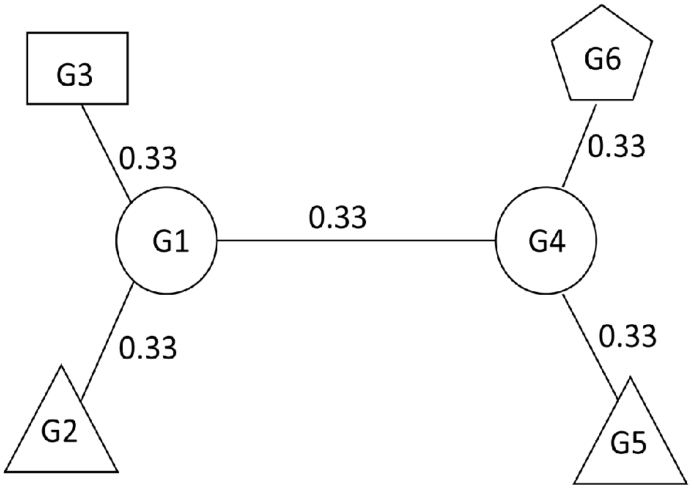

Phenotype	Shape
P1	○
P2	△
P3	□
P4	⬠

Figure S1.1. Example of an 'unweighted' genotype network

Genotype Robustness

In order to calculate genotype robustness of G1 (R_{G1}), we first identify the neutral neighbour(s) of G1: {G4}.

New definition: Probability of evolving to a neutral neighbour, R_{G1} = 0.33

Old definition: Fraction of neutral neighbours in 1-neighbourhood, R_{G1} = 1/3 =0.33.

We see that the values as given by the two definitions are identical.

Genotype Evolvability

We calculate the genotype evolvability of G1 (E_{G1}) as (**new definition**) the mean probability of evolving from G1 (with phenotype P1) to a different phenotype, summed over all the different phenotypes found in the 1-neighbourhood of the genotype.

In order to evaluate this, we first identify phenotypes different from G1 (forming phenotype P1) in the 1- neighbourhood of the genotype (G1): {G2 (P2)}, {G3 (P3)}, {G5 (P2)}.

Thus, number of unique structures in the 1-neighbourhood U = 2. Table S1.1 discusses the probabilities and occurrences of these unique structures in the 1-neighbourhood.

Phenotype in 1-neighbourhood	Total probability of evolving to the structure	Number of times the structure occurs	Mean probability of evolving to the structure
P2	0.66	2	0.33
P3	0.33	1	0.33

Table S1.1. Probabilities and occurrences of unique structures in the 1-neighbourhood of G1.

Finally, we define genotype evolvability as,

$E_{G1} = \sum_{1}^{U} \text{Mean probability of evolving to a different structure} = 2\times0.33 = 0.66$

Old definition:

Genotype evolvability = Fraction of unique structures in 1-neighbourhood of G1

E_{G1}= {P2, P3}/{P1, P2, P3} = 2/3 = 0.66.

Here also we observe that the two definitions yield identical values.

Phenotype Robustness

Phenotype robustness of P1 (R_{P1}):

We define phenotype evolvability as (**new definition**) probability of evolving to a neutral neighbour from a genotype averaged over all the genotypes with phenotype P1

In order to calculate this value, we first identify all genotypes forming phenotype P1: G1 and G4

Phenotype robustness is therefore given by average genotype robustness of G1 and G4

R_{P1} = (0.33+0.33)/2 = 0.33.

According to the **old definition,** phenotype robustness is the fraction of neutral neighbours of a genotype, averaged over all genotypes that form a given phenotype.

$R_{P1} = (R_{G1} + R_{G4})/2 = (\frac{1}{3} + \frac{1}{3})/2 = 0.33$.

We observe that the values resulting from both definitions are identical.

Phenotype evolvability

Phenotype evolvability of P1 (E_{P1}):

We calculate phenotype evolvability (**new definition**) as the mean probability of evolving to a different phenotype (structure), summed over all unique phenotypes in the 1-neighbourhood of P

For this, we first identify phenotypes different from P1 in its 1- neighbourhood: G2 (P2), G3 (P3), G5 (P2), G6 (P4).

Thus, the number of unique structures in the 1-neighbourhood of P1 is U = 3. Table S1.2 discusses the probabilities and occurrences of these unique structures in the 1-neighbourhood of P1.

Phenotype in 1-neighbourhood	Total probability of evolving to the structure	Number of times the structure occurs	Mean probability of evolving to the structure
P2	0.66	2	0.33
P3	0.33	1	0.33
P4	0.33	1	0.33

Table S1.2. Probabilities and occurrences of unique structures in the 1-neighbourhood of P1.

Finally, phenotype evolvability is defined as,

E_{P1}= $\sum_1^U$ Mean probability of evolving to a different structure = 3×0.33 = 1

Old definition: Number of unique phenotypes found in the 1-neighbourhood of the phenotype P1:

E_{P1} = U = {P2, P3, P4} = 3

To summarise, the values of the robustness and evolvability as given by both the new and old definitions are:

Property	Value (New definition)	Value (Old definition)
Genotype robustness R_{G1}	0.33	0.33
Genotype evolvability E_{G1}	0.66	0.66
Phenotype robustness R_{P1}	0.33	0.33
Phenotype evolvability E_{P1}	1 (= 3×0.33)	3

Table S1.3. Values of the robustness and evolvability (both new and old definitions).

We see that if all edges have the same probability (*p*), as in an unweighted network, then

$\frac{\text{phenotype evolvability (new definition)}}{\text{phenotype evolvability (old definition)}}$ = *p*, a constant.

In the above example network, *p* =0.33.

We observe that both the new and old definitions of genotype robustness and evolvability and phenotype robustness yield identical values for an unweighted network. The new definition of phenotype evolvability essentially reduces to the earlier definition, only scaled by a constant value, in the case of an unweighted network.

Weighted Neutral Networks

Let us now consider a weighted network with the same genotypes and phenotypes. However, the probabilities corresponding to different mutations are no longer assumed to be the same.

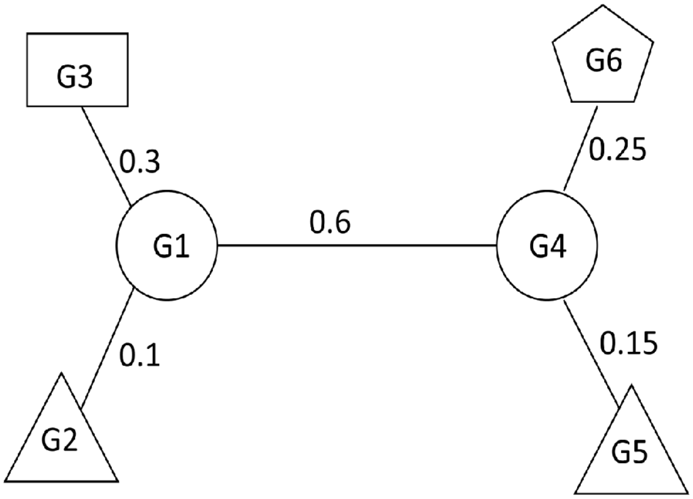

Phenotype	Shape
P1	○
P2	△
P3	□
P4	⬠

Using the above definitions, we calculate the values of robustness and evolvability for this weighted network:

Property	Value (New definition)	Value (Old definition)
Genotype robustness R_{G1}	0.6	0.33
Genotype evolvability E_{G1}	0.133	0.66
Phenotype robustness R_{P1}	0.6	0.33
Phenotype evolvability E_{P1}	0.625	3

Table S1.4. Values of robustness and evolvability for the weighted network.

We see that weighting the network essentially does not change the values of robustness and evolvability (both genotype and phenotype), according to the earlier (old) definitions. Thus, we modified these definitions, and came up with a new set of definitions for the system properties that tries to incorporate edge (mutation) probability. Also, for an unweighted network, these modified definitions of robustness and evolvability yield identical values to those given by the earlier definitions.

References

1. Wagner A (2008) Robustness and evolvability: a paradox resolved. Proc Biol Sci 275: 91–100. Available: http://www.pubmedcentral.nih.gov/articlerender.fcgi?artid=2562401&tool=pmcentrez&rendertype=abstract. Accessed 28 February 2013.

2. Gruber AR, Lorenz R, Bernhart SH, Neuböck R, Hofacker IL (2008) The Vienna RNA websuite. Nucleic Acids Res 36: W70–4. doi:10.1093/nar/gkn188.
